# Hair today, gone tomorrow: How personal protective equipment guidance changed doctor's facial hair during the COVID‐19 pandemic

**DOI:** 10.1002/hsr2.278

**Published:** 2021-05-07

**Authors:** Sanjeev Sahota, Simon Gill, Jennifer Ridenton, Helen Hegarty, Katherine Pope, Giorgio Gentile

**Affiliations:** ^1^ Brighton and Sussex University Hospitals NHS Trust, Anaesthetics Brighton and Hove UK; ^2^ Royal Cornwall Hospitals NHS Trust, Anaesthetics Truro UK; ^3^ Royal Cornwall Hospitals NHS Trust Cardiology Truro UK; ^4^ Sussex Partnership Trust, Department of Psychiatry Eastbourne General Hospital Eastbourne UK; ^5^ Royal Cornwall Hospitals NHS Trust Paediatrics Truro UK; ^6^ Royal Cornwall Hospitals NHS Trust Nephrology Truro UK; ^7^ University of Exeter, Medicine, The Knowledge Spa Exeter Devon UK

**Keywords:** beard, COVID 19, facial hair, FFP3, mask, moustache, N95, occupational health, personal protective equipment, public health, wellbeing

## Abstract

**Objectives:**

To investigate how personal protective equipment (PPE) guidance altered the facial hair of hospital doctors and explore the wider impact and implications of these changes.

**Methods:**

A single site uncontrolled before‐after survey study examining change in facial hairstyles, and wider implications on doctor's cultural, religious, and personal wellbeing. Outcome measures included change in facial hair between January and April 2020 and whether these changes adhered to guidance set by Public Health England. Participants were also asked about the wider impact of these changes which were thematically analyzed using an inductive approach.

**Results:**

Of those who completed the survey, 257 participants met the inclusion criteria. 68% (n = 67) of doctors who could grow facial hair changed their facial hairstyle during the COVID‐19 pandemic and 96% (n = 64) reported that the change was in response to PPE guidance. The odds of having a facial hairstyle that complied with PPE guidance before the pandemic was 0.32, which rose to 2.77 after guidance was released, giving an odds ratio of 8.54 (95% CI 4.49‐16.23, *P* < .001). When compared to those who sported a shaven face prepandemic, the odds ratio of a change in style for those with prepandemic full beards was 37.92 (95% CI 7.45‐192.8, *P* < .001), for goatees was 7.22 (95% CI 1.076‐48.47, *P* = .04), for moustaches was 4.33 (95% CI 0.207‐90.85, *P* = .345), and for stubble was 9.06 (95% CI 2.133‐38.49, *P* = .003). Qualitative analysis revealed multiple themes, including skin irritation, loss of identity, and a significant impact on participants required to maintain a beard due to religious or cultural reasons.

**Conclusions:**

Facial hairstyles have changed significantly at our hospital during the COVID‐19 pandemic. Facial hair can impact upon doctors' cultural, religious, and personal wellbeing and these factors need to be considered with policy and provision of PPE.

## 1 INTRODUCTION

The Coronavirus disease 2019 (COVID‐19) global pandemic constitutes the biggest public health crisis since the 1918 “Spanish flu.” COVID‐19 is caused by the RNA coronavirus SARS‐CoV‐2, which is transmitted in a number of ways: by droplets, direct contact with infected patients, fomites (contaminated items), or during procedures which involve aerosolization.^1^, ^2^, ^3^ As such, over the past year, many changes have been made to the way we live our lives to prevent transmission of COVID‐19, including social distancing, increased hand hygiene, and diligent cleaning of surfaces.^3^, ^4^, ^5^ These protective measures have also included the wearing of masks, which can be very broadly categorized into surgical masks and respirators. Surgical masks are designed to reduce person‐to‐person transfer of droplets and are designed to protect those surrounding the wearer.^6^, ^7^, ^8^ Respirators on the other hand are tight‐fitting masks designed to create a seal around the nose and mouth, preventing the inhalation of very small particles and therefore protect the wearer themselves.^6^, ^7^

**FIGURE 1:**
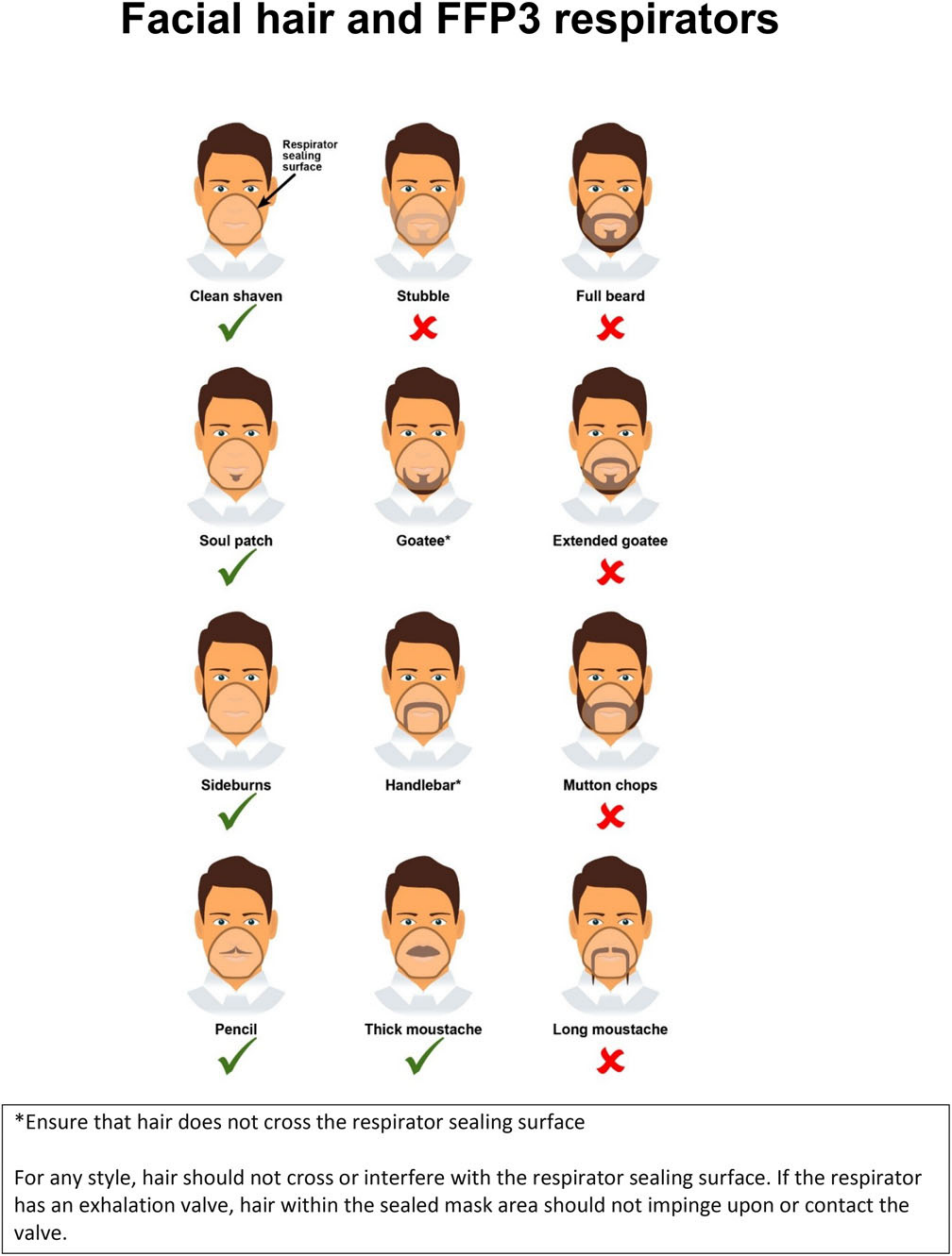
Public Health England guidance on acceptable facial hairstyles

Healthcare workers are a high risk group of individuals for occupational exposure to SARS‐CoV‐2. It is possible to contract COVID‐19 from close contact of confirmed or suspected patients, which often occurs before knowing the patient's COVID‐19 status.^3^ In response, all healthcare workers in the United Kingdom are required to wear personal protective equipment (PPE) in the clinical environment both to reduce transmission and to protect themselves.^9^ This may include the use of tight‐fitted Filtering Face Piece class 3 (FFP3) masks for aerosol‐generating procedures (AGPs). Studies have identified an association between FFP3 mask leakage and the presence of facial hair.^10^, ^11^ In accordance with this data, Public Health England (PHE) and the Health and Safety Executive (HSE) advise all healthcare workers to undergo “fit testing” and advise that facial hair does not cross the mask's sealing surface in order to minimize leakage^9^ as there is evidence that improper fit of respirators may have caused healthcare workers to contract COVID‐19.^12^ PHE have issued an infographic adapted from guidance by the Centres for Disease Control (CDC) on acceptable facial hair for FFP3 masks (Figure 1).^13^

Prior to COVID‐19, it is the authors' experience that there had been a considerable increase in the number of hirsute colleagues, sporting what were colloquially known as, “hipster beards,” in recent years. Despite growing trends in facial hair in the general population during lockdown and recent articles in men's magazines on “How to Grow a Beard in Style,”^14^ facial topiary within medicine has come under close scrutiny during the coronavirus pandemic. Facial grooming has played many roles relating to health and risk of infection throughout history. In the sixteenth century, Pierus Valerianus argued that a beard preserved the teeth from rotting and defended “the face from the burning rays of the sun,” a claim supported by Parisi et al in 2011, five centuries later.^15^, ^16^ Many Victorians heralded men with beards; from early aspirational bearded explorers being encouraged to grow facial locks to filter out germs to links between long beards and the hardiness of wearers being “less subject to diseases”.^16^ In the early 20th century, beards became a scapegoat for infection and people were encouraged to “Shave the Microbe Infested Beard” to prevent the spread of disease from “a well‐whiskered face.”^17^ It seems that it is not only humans that have a predilection for certain grooming styles, as is suggested by the preference of the microbes *Staphylococcus aureus* and MRSA to colonize clean‐shaven healthcare workers over their bearded counterparts.^18^ However, studies have also demonstrated that bearded men shed bacteria more readily, even with masks, and that bacteria remain on, and are more difficult to remove from beards, even with intense washing.^19^, ^20^ That said, further research has refuted this, showing no difference in bacterial shedding between bearded and clean‐shaven subjects, although a nonsignificant trend to shed more bacteria was seen, and this trend terminated with addition of a surgical mask.^21^

Infection prevention and control aside, there are many complex personal and social reasons why people grow beards, including religious and cultural commitments, for example in Sikhism. Additionally, research in the field of sociology suggests that beards may also convey competency, attractiveness, and maturity.^22^, ^23^, ^24^

This study investigated how PPE guidance changed the facial hair of doctors working at a teaching hospital and whether these changes adhered to guidance set by PHE, while exploring the wider impacts and implications of these changes on individuals.

## 2 METHODS

The Facial Adaptations Caused by Instructions Around Lockdown: Hair maintenance And other Interesting Ramifications (FACIAL HAIR) study was a single site uncontrolled before‐after survey study which compared the facial grooming habits of hospital doctors before and after the aforementioned PPE guidance was published, and explored their thoughts and feelings relating to their facial furnishings and to PPE. Data were collected with a survey incorporating a mixed‐methods approach using both open and closed questions, and a triangulation design. Participants were all doctors working at the Royal Cornwall Hospital (RCH). A list of 956 doctors working at RCH at the time was provided by the postgraduate center and an email with an information sheet and a link to the survey (powered by Survey Monkey) was distributed. The survey enquired about facial hairstyles prior to the first confirmed the UK case of COVID‐19 on January 30, 2020^25^ and after commencement of a national lockdown enforced by the UK Government on March 23, 2020.^26^ To ensure maximal inclusion, and avoid discrimination, hospital doctors of all genders and demographics were invited to take part.

### 2.1 Inclusion criteria

All hospital doctors working at the main RCH site between January and April 2020 were included in the study population.

### 2.2 Exclusion criteria

Those excluded were doctors not currently working at the main RCH site during the study period, including those on rotations in the community, to remove the effect of any variances in PPE guidance. While not excluded from the whole study, those who were unable to grow facial hair were not included in parts of the statistical analysis pertaining to the choice of facial hair.

### 2.3 Outcome measures

The primary outcome measure was self‐reported change in facial hairstyle of facial hair‐growing respondents between January and April 2020. Secondary outcome measures included whether there was a change in the proportion of PPE‐adherent facial hair styles, and whether initial facial hairstyle, age, grade, speciality, and involvement in AGPs affected this change. All study participants were asked their preferred style of facial furnishing and were given the opportunity to provide additional comments on any aspect of facial hair and PPE during COVID‐19. Due to limitations to the number of diagrams that are permitted in questions on Survey Monkey, and to increase statistical power, some facial hairstyles were combined. Handlebar, pencil, thick, and long moustaches were grouped into “moustaches which did not cross chin/jaw” (i.e., guidance adherent) and “moustaches which did cross chin/jaw” (i.e., guidance nonadherent). The same was done for goatee and extended goatee splitting them into adherent and nonadherent groups. However, the original PHE categories were used when asking participants about their favorite guidance adherent facial hairstyle to capture people's preferences more accurately.

Open‐ended questions in the questionnaire, where participants were invited to provide free‐text responses, are outlined below:

What was the impact on changing your facial hair style?

Has this affected your mental health and wellbeing?

Do you have any religious or cultural reasons for maintaining any facial hair style?

What is the religious/cultural impact if you were to not maintain this facial hair style?

Do you have any other comments about FFP3 masks and facial hair guidance?

### 2.4 Statistical analysis

For the quantitative analysis, odds ratios were calculated using R version 4.0.2 (R Foundation for Statistical Computing, Vienna, Austria) for a change in facial hair by age, grade, clinical speciality, and prepandemic facial hairstyle subgroups. Odds ratios were also calculated for PPE‐compliant hairstyles pre‐ and post‐pandemic. Thematic analysis was undertaken for qualitative data, using an inductive approach whereby responses were coded by ideas and feelings expressed, collated into groups, and combined into prevailing themes. A triangulation approach was used to integrate quantitative and qualitative data to give a better understanding of the thoughts and feelings associated with the outcomes observed.

## 3 RESULTS

303 doctors responded to the survey. A total of 46 were excluded (20 did not work at the main RCH site, 2 did not consent, 12 were interim foundation doctors who were not working at the trust in January 2020, and 12 did not complete the survey) leaving 257 participants. Of these there were 100 females, 154 males, none who described their gender as other and three who did not disclose their gender. 104 respondents were able to grow facial hair of whom 98 gave details of facial hairstyle pre‐ and post‐PPE guidance. Table 1 shows the baseline characteristics of participants able to grow facial hair.

**TABLE 1:** Baseline characteristics of participants able to grow facial hair

Category	Subgroup, (sample size, n)	Change in facial hair (number, %)		No change in facial hair (number, %)	
Grade	Foundation (n = 18)	10	56%	8	44%
	CT (n = 17)	13	76%	4	24%
	SAS (n = 8)	5	63%	3	38%
	ST (n = 17)	14	82%	3	18%
	Consultant (n = 39)	25	64%	14	36%
Speciality	Acute GP (n = 1)	0	0%	1	100%
	Anesthetics (n = 18)	14	78%	4	22%
	EM (n = 5)	4	80%	1	20%
	Medicine (n = 34)	21	62%	13	38%
	Obstetrics (n = 2)	1	50%	1	50%
	Oro‐facial surgery (n = 4)	3	75%	1	25%
	Pediatrics (n = 7)	5	71%	2	29%
	Radiology (n = 2)	0	0%	2	100%
	Surgery (n = 20)	14	70%	6	30%
	Other (n = 6)	5	83%	1	17%
Age	20‐30 (n = 26)	17	65%	9	35%
	30‐40 (n = 31)	22	71%	9	29%
	40‐50 (n = 23)	16	70%	7	30%
	50+ (n = 18)	11	61%	7	39%
	Undisclosed (n = 1)	1	100%	0	0%
AGP exposure	Yes (n = 87)	60	69%	27	31%
	No (n = 12)	7	58%	5	42%
PPE level	1 (n = 4)	1	25%	3	75%
	2 (n = 68)	44	65%	24	35%
	3 (n = 27)	22	81%	5	19%

*Note*: CT, core trainee; SAS, staff grade and associate specialist; GP, general practitioner; EM, emergency medicine; AGP, aerosol‐generating procedure, Level 1 PPE = minimal or no PPE, Level 2 PPE = surgical fluid resistant mask, gloves and disposable apron, Level 3 PPE = FFP3 mask or hood, long sleeve gown, long sleeve gloves, eye protection).

68% (n = 67) of respondents who were able to grow facial hair changed their facial hairstyle between January and April 2020; of those, 96% (n = 64) reported that this change was influenced by PPE guidance. Notably, of the 39 once fully bearded doctors, only four retained their formerly hirsute state. Of those able to grow a beard, 14 remained clean shaven throughout the study period, and 43 became newly clean shaven. Fifty‐three men reported that they were unable to grow significant facial hair amenable to styling. Of note, all moustaches throughout the entire study period adhered to PPE guidance and did not cross the jaw or chin. When compared to those who sported a clean‐shaven face in January 2020, the odds ratio of a change in style for those with full beards was 37.92 (95% CI 7.45‐192.8, *P* < .001), for goatees (both crossing chin and not crossing chin) was 7.22 (95% CI 1.076‐48.47, *P* = .04), for moustaches was 4.33 (95% CI 0.207‐90.85, *P* = .345) and for stubble was 9.06 (95% CI 2.133‐38.49, *P* = .003). Table 2 shows a grid of facial hairstyles in January compared to April 2020 (N.B. Of the 39 participants with beards in January, one did not disclose their April facial hairstyle and therefore has been omitted from Table 2). Table 3 shows demographic and fit test data in relation to participants who do and do not perform AGPs.

**TABLE 2:** Hair grid showing participant's pre and post styles

		Facial hairstyle April 2020 (n = 98)							
		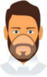	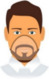	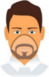	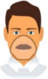	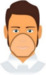	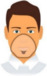	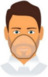	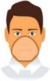
		Full beard (n = 4)	Goatee (CC)(n = 2)	Goatee (NCC) (n = 6)	Moustache (NCC) (n = 7)	Sideburns (n = 1)	Soul patch (n = 1)	Stubble (n = 20)	Shaven (n = 57)
Facial hairstyle January 2020 (n = 98)	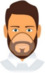 Full beard, (n = 38)	4 (10.5%) **[0.11]**	1 (2.6%) **[0.03]**	3 (7.8%) **[0.08]**	2 (5.2%) **[0.05]**	1 (2.6%) **[0.03]**	0 (0%) **[0]**	6 (15.7%) **[0.16]**	21 (55.2%) **[0.55]**
	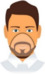 Goatee (CC) (n = 2)	0	2 (50%) **[0.5]**	0	0	0	0	0	2 (50%) **[0.5]**
	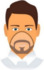 Goatee (NCC) (n = 6)	0	0	2 (25%) **[0.33]**	1 (12.5%) **[0.17]**	0	0	0	3 (50%) **[0.5]**
	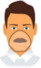 Moustache (NCC) (n = 2)	0	0	0	1 (50%) **[0.05]**	0	0	0	0
	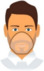 Stubble (n = 34)	0	0	0	3 (8.8%) **[0.09]**	0	1 (2.9%)**[0.03]**	12 (35.2%) **[0.35]**	18 (52.9%)**[0.53]**
	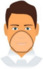 Shaven, (n = 16)	0	0	0	0	0	0	2 (12.5%)**[0.13]**	14 (87.5%) **[0.88]**

*Note*: CC denotes “crossing chin,” NCC denotes “not crossing chin.” Total number is outside brackets, percentage of new facial hairstyle compared to number with facial hairstyle in same row in brackets (eg, full beard post compared to full beard pre is 4 divided by 38 as percentage), and risk ratio is below, in bold square brackets. Shaded boxes indicate no change in facial hairstyle. Total number = 98 rather than 99 due to 1 participant disclosing only their January facial style. No participants had sideburns or a soul patch in January, so these rows have been omitted. No participants had a moustache which crossed the chin or jaw, so these rows and columns have been omitted. Images have been adapted from PHE guidance on facial hair^4^ (reprinted with permission).

**TABLE 3:** Demographic and fit test data relating to performance of aerosol generating procedures (AGPs)

All participants (total males = 153, total females = 99)									
Perform AGP	Males who can grow facial hair			Males who cannot grow facial hair			Females		
	89			49			81		
	All males = 138								
	All genders = 219								
Do not perform AGP	Males who can grow facial hair			Males who cannot grow facial hair			Females		
	12			3			18		
	All males = 15								
	All genders = 33								
	*Passed fit testing*			*Failed fit testing*			*Not tested*		
Perform AGP	Males who can grow facial hair	Males who cannot grow facial hair	Females	Males who can grow facial hair	Males who cannot grow facial hair	Females	Males who can grow facial hair	Males who cannot grow facial hair	Females
	73 (82.02%)	42 (85.71%)	68 (83.95%)	10 (11.24%)	5 (10.20%)	13 (16.04%)	6 (6.74%)	2 (4.08%)	0
	All males = 115 (83.33%)			All males = 15 (10.87%)			All males = 8 (5.80%)		
	All genders = 183 (83.56%)			All genders = 28 (12.79%)			All genders = 8 (3.65%)		
Do not perform AGP	Males who can grow facial hair	Males who cannot grow facial hair	Females	Males who can grow facial hair	Males who cannot grow facial hair	Females	Males who can grow facial hair	Males who cannot grow facial hair	Females
	9 (75%)	2 (66.67%)	8 (44.44%)	0	0	2 (11.11%)	3 (25%)	1 (33.33%)	8 (44.44%)
	All males = 11 (73.33%)			All males = 0			All males = 4 (26.67%)		
	All genders = 19 (57.58%)			All genders = 2 (6.06%)			All genders = 12 (36.36%)		
	Total males passed = 126 (82.35%)			Total males failed = 15 (9.8%)			Total males not tested = 12 (7.84%)		
	Total females passed = 76 (76.77%)			Total females failed = 15 (15.15%)			Total females not tested = 8 (8.08%)		

*Note*: Total number is outside the bracket, and percentage of those compared to total is inside the bracket. The three participants who did not disclose their gender, and two others with incomplete AGP and/or fit test data have been omitted from this table.

There was a statistically significant change in PPE‐adherent facial hairstyles. The odds of having a facial hairstyle that complied with PPE guidance in January was 0.32, which rose to 2.77 in April, after guidance was released, with an odds ratio of 8.54 (95% CI 4.49‐16.23, *P* < .001).

There were no statistically significant differences in changing facial hairstyle across age, grade, or speciality. It is noted however that none of the radiologists who completed the survey changed their facial hair compared to 78% of Anesthetists, 80% of Emergency Medicine doctors, 62% of Physicians, 70% of Surgeons, 71% of Pediatricians, and 75% of Oro‐facial surgeons.

69% of those exposed to AGPs changed their facial hair (odds 2.22) vs 58% of those who were not exposed (odds 1.4). The odds ratio in those exposed vs not exposed was 1.59 (95% CI 0.46‐5.45, *P* = .463). Of the 231 respondents who reported their preferred PPE‐adherent style, a clean‐shaven face was the clear winner, favored by 65% of respondents (n = 150; 71% of women and 61% of men). In second place came the goatee (n = 35; 15% overall, 14% of men and 17% of women). This was followed by the thick moustache (n = 17; 7%), sideburns (n = 12; 8%), pencil moustache (n = 7; 3%), and handlebar moustache (n = 7; 3%). In last place was the soul patch at 1% (n = 3). That said, the one person who adopted this style during the pandemic was happy with their new look giving it a 100% satisfaction rate, compared to 48% (n = 21) of the freshly shaven, 44% (n = 4) of the newly stubbled, and 40% (n = 2) of those with a recent goatee.

Thematic analysis of free text responses revealed some recurring themes. When asked about the impact of changing their facial hairstyle, the most prevalent issues reported by our respondents were skin irritation and the increased time spent shaving. Many disliked their new appearance; with some feeling they had lost a defining characteristic and others reporting that they had not been recognized by family and colleagues. Some reported feeling discriminated against; with one doctor explaining how, despite passing fit testing with his beard, he had still felt pressurized to shave. On the other hand, some found themselves and their partners preferring their new style, many relishing their more youthful appearance. Table 4 shows some quotes from the free text responses.

**TABLE 4:** A selection of the free text responses

“*Apart from losing what I felt was a defining part of my appearance, I now feel that I look much younger*.”
“*A lot of my identity is attached to my beard, I've had it since the age of 18 and don't recognise the person in the mirror at the moment*.”
“*Had to re‐learn to use a razor. Picked up a few nicks and cuts!*”
“*Stubble usually present and hides scarring from teenage acne ‐ so element of psychological impact of wet shaving there*.”
“*I want my beard back actually. I don't like my face without my beard*”
“*Gut wrenching change*.”
“*Bit weird as first time without a full beard in 10 years. I quite like it, mixed comments from colleagues!*”
“*My partner of 3 years has never seen me clean‐shaven before and doesn't really like it!*”
“*Physical – skin irritation from daily shaving. Psychological ‐ dysphoric sense of self*”
“*Physically I started to suffer with folliculitis and facial itching secondary to the fact that I am not used to shaving that often on a daily basis*”
“*I feel self‐conscious without it*”
“*In my culture, a moustache is a sign of manhood*”
“*Religion (Islam) allows that if in extreme circumstances or life‐saving circumstances that one must remove… however I would rather keep my beard especially knowing that alternatives (i.e. hoods) do exist even if I have to make the financial commitment. I know many other Muslim colleagues who feel this way*.”
“*Some colleagues have reported that they can't take me seriously with my new look, but I have a facemask on most of the time so that's negated*”
“*My children didn't recognise me. Neither did my phone*.”
“*Negative impact on my wellbeing as I'd been growing it hard for a few years*”
“*I've become a 12‐year‐old boy again*.”

Six respondents reported religious or cultural reasons for maintaining their facial hairstyle. Of these, one had been offered a hood and the remaining five changed their facial hair in line with PPE guidance. None of them reported feeling happy with their new style. One respondent commented:

Growing my beard is an Islamic duty and a code of our identity…I feel an alternative and beard friendly PPE should be looked into.

## 4 DISCUSSION

**FIGURE 2:**
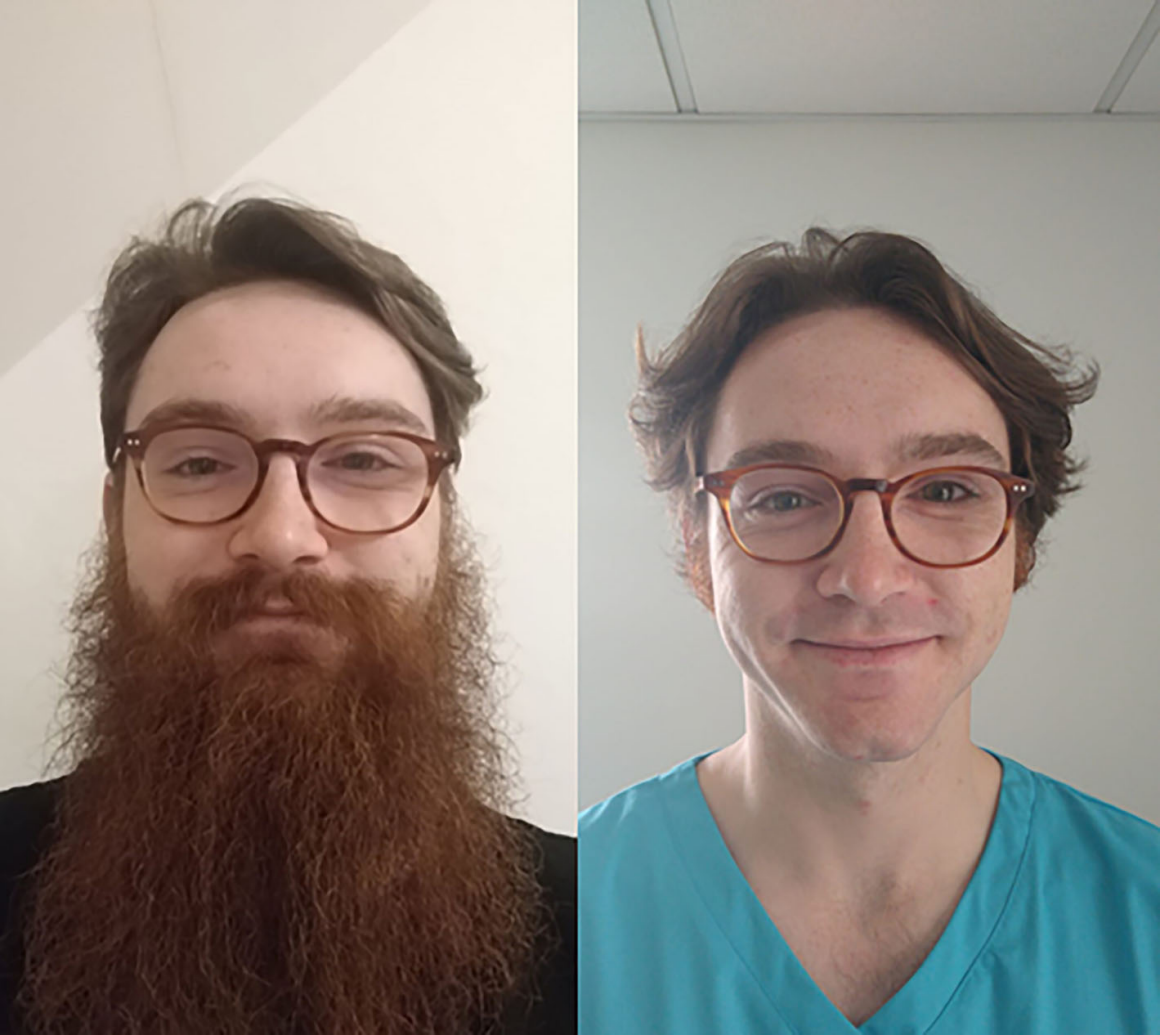
A junior doctor who has changed their facial hairstyle during the pandemic (printed with consent)

This study found that facial hair has significantly changed at RCH during the COVID‐19 pandemic, with a move away from beards toward clean shaves (Figure 2). To the authors' knowledge, this is the first study to assess the effect of PPE guidance on facial hair during the COVID‐19 pandemic. Facial topiary has long sparked controversy in the spheres of fashion, politics, and hygiene, and has been targeted during past pandemics.^17^, ^18^, ^27^, ^28^ Most recently, the NHS has recommended that Trusts ask their staff if they would be willing to be clean‐shaven in order to ensure optimal fit of FFP3 respirator masks.^29^ However, this study suggests that a clean‐shaven face does not necessarily correlate to passing fit testing. Of the 30 that failed, only nine could grow facial hair of which three had noncompliant styles while of the 26 that had noncompliant styles in April, only three failed fit testing (5 not tested, 18 passed). Additionally, a greater proportion of men (82.35%) passed than women (76.77%), supporting some publicly expressed concerns that PPE is designed to be worn by Caucasian males despite 45% of the NHS doctor workforce being female and 27% of doctors being non‐Caucasian in 2019.^30^, ^31^, ^32^ In fact, a recent study confirmed this anecdotal evidence with 18.2% of women failing fit testing compared to 9.7% of men at a UK hospital (*P* < .01), and our results mirror this.^33^

Although no statistical significance was found in changing facial hairstyle across age, grade, or speciality, there are some trends of note, with more anesthetists changing facial hair than their surgical or medical colleagues, and none of the radiologists involved in the study changing their facial grooming style at all. Reasons for this lie beyond the scope of this study; however, this may be associated with exposure to patients and AGPs. There have been many changes and challenges that certain specialties have had to undertake relating to COVID‐19. In the field of anesthetics, alongside routine use of PPE, there have been many alterations to practice, such as avoiding elective intubations, using intubation boxes as barriers, and employing different techniques to face‐mask ventilate patients, as well as procedural changes such as using negative pressure rooms and having only essential personnel present.^34^, ^35^, ^36^ From a surgical perspective, there have also been changes to practice such as fewer laparoscopies, more exploratory laparotomies, and use of alternative techniques to create surgical airways.^37^, ^38^, ^39^ Additionally, for surgeons there have been other implications of using PPE, with evidence that using PPE affects performance, reduces comfort, increases difficulties in communication, and impairs decision making processes and situational awareness.^40^ We speculate that these changes to practice in these specialities may have increased awareness of the importance for all staff to pass fit testing. This may provide some insight into why there was a small trend for more theatre staff such as anesthetists and surgeons to change their facial hair, compared to GP's and radiologists, however, the numbers in this study were too small to make generalizations and further investigation with increased statistical power would be required to draw more definitive conclusions.

Our study also highlights some personal ramifications on shaving facial hair, both physical and emotional, including cultural and religious obligations. Comments from doctors in the study raise important concerns regarding the lack of alternative PPE provided, which should be considered in light of NHS employers' guidance on equality and diversity.^41^ The implications of the impact that certain items of PPE may have on religious beliefs and vice versa cannot be understated, especially as mounting evidence shows that those from Black and Minority Ethnic (BAME) groups are disproportionately affected by COVID‐19.^42^ Facial hair is also sociologically complex, and it is clear that it is an integral part of many people's identity. Although it is traditionally associated with ideas of masculinity, as society progresses beyond a binary view of gender, ideas such as this are evolving and becoming much more complex and nuanced. Respondents commonly reported that they felt younger without their facial hair and some reported that they felt their colleagues could not take them seriously or even recognize them with their new style. Another theme from the qualitative analysis was of doctors' romantic partners' preference for their facial hair habits, with some preferring facial hair, and others relishing their new looks.

With evidence now emerging that at least one in five healthcare professionals have reported mental health issues during the COVID‐19 pandemic, it is more important now than ever to consider the welfare and well‐being of our frontline staff.^43^ The physical, mental, and social well‐being of the workforce precedes good clinical care, and as such, though seemingly innocuous, facial hair has been shown by our responses to be an important aspect of personal well‐being.^44^ Guidance recommends employers balance the psychological impact on the individual against the health and safety implications and consider alternative PPE or staff redeployment if required.^29^

### 4.1 Limitations of this study

This study has several limitations. It is difficult to know precisely how many doctors were working at RCH due to frequent movement of the workforce during training rotations, particularly to those healthcare providers outside of the hospital. In our experience, NHS mailing lists are often out‐of‐date, and in an organization as large as an NHS hospital, these generic mailing lists often significantly over‐estimate the number of doctors currently working. The time of this study was also during the peak of the first wave of COVID‐19, and so our participants, all doctors, would have likely had higher priorities than complete our survey, although we announced the study at all appropriate meetings and in regular multiple email reminders. However, our estimated response rate of 32%, although likely higher in reality, is an acceptable response rate for this type of research, and we closed our survey once there were no more responses over a 2 week period.^45^ Participants completing the questionnaire were self‐selecting, and therefore may have had biases that prompted them to complete the survey. Therefore, our respondents may not be representative of an ordinary sample of doctors and could over‐represent those who were motivated to change their facial hair. In addition, the questionnaire‐based method is not as reliable as conducting verbal interviews with participants; however this would have greatly limited numbers. Also, the categorization of beards from PHE has limited options and may not have captured the diversity of specific facial hairstyles that doctors chose to adopt. The original CDC chart, circulated on social media in March 2020, has a total of 36 different hairstyles. The PHE adaptation of this was chosen as this is UK guidance and the reduced number of options increases the statistical power of the study.^46^ Another drawback is that demographically the population of Cornwall is predominately Caucasian with only 1.8% identifying as a nonwhite ethnic group in the most recent 2011 census. Therefore, although the study identified issues around facial hairstyle, religious, and other beliefs, they may not be applicable to other areas of the United Kingdom. That said, although participants were not specifically asked to disclose their ethnicity in the questionnaires, anecdotally there is a higher percentage of non‐Caucasian people in the RCH workforce than live in the general population in the area.

### 4.2 Implications of this study

Recent PPE guidance has clearly had an effect on the facial hair of doctors at RCH. Our analysis of the qualitative data show that this has had some negative impact from an equality and diversity perspective, as well as on a personal level for some. As facial hair is not the only factor involved in the fit of FFP3 masks, the negative impact of the guidance may be larger than anticipated. Various shapes of FFP3 mask have been designed, in addition to other PPE such as hoods. Our study supports the use of the right PPE for each individual person, rather than a generalized approach. This of course may be difficult with the financial constraints of the NHS, but this does not negate the need for it. There is a requirement for further research in this area to fully identify the impact of PPE guidance and its effect on diversity and identity of the workforce as well as to promote work to ensure a broader range of available PPE designs for future use in healthcare. This is essential in order to ensure the safety and well‐being of all NHS employees.

## 5 CONCLUSIONS

Facial hairstyles have changed significantly at this hospital during the COVID‐19 pandemic. It is important during this difficult time that staff work together and support each other as best as possible to increase patient safety while respecting others' beliefs and preferences. As almost every aspect of normal life has been disrupted over the past year, it appears that even the seemingly innocuous “hipster beard” has fallen foul of the COVID‐19 pandemic.

## FUNDING

The study received no external or internal funding from any source.

## CONFLICT OF INTEREST

The authors have no financial or pecuniary conflicts of interests to disclose. Other non‐financial relevant interests: all authors abstained from taking part in the survey for impartiality. However, though previously full bearded, Sanjeev Sahota is particularly fond of the thick moustache in the style of Freddie Mercury and this has been his PPE adherent facial hair of choice during the pandemic. Simon Gill has a soft spot for the soul patch, which he temporarily sported as a throwback to his skater past. Jennifer Ridenton prefers sideburns, Katherine Pope is a big fan of the handlebar moustache for reasons she refuses to disclose, and against her better judgment Helen Hegarty greatly appreciates the full goatee despite her genuine frustration about not being able to grow one. Giorgio Gentile abstained from these discussions for impartiality.

## AUTHOR CONTRIBUTIONS

All authors meet ICMJE criteria for authorship.

Conceptualization: Sanjeev Sahota, Simon Gill, Jennifer Ridenton, Helen Hegarty

Data Curation: Katherine Pope

Formal Analysis: Katherine Pope, Giorgio Gentile

Investigation: All authors

Methodology: All authors

Project Administration: Sanjeev Sahota

Software: Katherine Pope, Giorgio Gentile

Supervision: Giorgio Gentile

Validation: Giorgio Gentile.

Visualization: Katherine Pope, Sanjeev Sahota

Writing—Original Draft Preparation: Sanjeev Sahota

Writing—Review and Editing: All authors

All authors have read and approved the final version of the manuscript.

Giorgio Gentile had full access to all of the data in this study and takes complete responsibility for the integrity of the data and the accuracy of the data analysis.

## TRANSPARENCY STATEMENT

Giorgio Gentile affirms that the manuscript is an honest, accurate, and transparent account of the study being reported; that no important aspects of the study have been omitted; and that any discrepancies from the study as planned have been explained.

## ETHICS STATEMENT

As the study was aimed at NHS staff and involved the completion of an anonymized, online questionnaire, the regulatory documents were completed and issued to the governing bodies, but it was confirmed that the project did not require an ethics review. However, the project was submitted to the Health Research Authority (HRA) for review and it received approval on the 13th May 2020 (IRAS ID 283609, REC Reference 20/HRA/2470). All participants were hospital staff who received a participant information sheet and gave their written informed consent before taking part.

## Data Availability

The full dataset is available upon request. Requests for access to data should be addressed to Giorgio Gentile.
